# Human Cytomegalovirus Immediate Early Protein 2 Protein Causes Cognitive Disorder by Damaging Synaptic Plasticity in Human Cytomegalovirus-UL122-Tg Mice

**DOI:** 10.3389/fnagi.2021.720582

**Published:** 2021-11-01

**Authors:** Zhifei Wang, Wenwen Yu, Lili Liu, Junyun Niu, Xianjuan Zhang, Fulong Nan, Lili Xu, Bin Jiang, Dingxin Ke, Wenhua Zhu, Zibin Tian, Yashuo Wang, Bin Wang

**Affiliations:** ^1^Department of Pathogenic Biology, School of Basic Medicine, Qingdao University, Qingdao, China; ^2^Department of Pharmacology, School of Pharmacy, Qingdao University Medical College, Qingdao, China; ^3^Qingdao Women and Childrens Hospital, Qingdao University, Qingdao, China; ^4^Affiliated Hospital of Qingdao University Medical College, Qingdao, China; ^5^Qingdao Fuwai Cardiovascular Hospital, Qingdao, China; ^6^Department of Gastroenterology, The Affiliated Hospital of Qingdao University, Qingdao, China; ^7^College of Life Sciences, Qingdao University, Qingdao, China

**Keywords:** cognitive disorders, IE2, HCMV, synaptic plasticity, plasticity related proteins

## Abstract

Human cytomegalovirus (HCMV) infection is very common in the human population all around the world. Although the majority of HCMV infections are asymptomatic, they can cause neurologic deficits. Previous studies have shown that immediate early protein 2 (IE2, also known as UL122) of HCMV is related with the cognitive disorder mechanism. Due to species isolation, a HCMV-infected animal model could not be established which meant a study into the long-term effects of IE2 on neural development could not be carried out. By establishing HCMV-UL122-Tg mice (UL122 mice), we explored the cognitive behavior and complexity of neuron changes in this transgenic UL122 mice that could consistently express IE2 protein at different ages (confirmed in both 6- and 12-month-old UL122 mice). In the Morris water maze, cognitive impairment was more pronounced in 12-month-old UL122 mice than in 6-month-old ones. At the same time, a decrease of the density of dendritic spines and branches in the hippocampal neurons of 12-month-old mice was observed. Moreover, long-term potentiation was showed to be impaired in 12-month-old UL122 mice. The expressions of several synaptic plasticity-regulated molecules were reduced in 12-month-old UL122 mice, including scaffold proteins postsynaptic density protein 95 (PSD95) and microtubule-associated protein 2 (MAP2). Binding the expression of IE2 was increased in 12-month-old mice compared with 6-month-old mice, and results of statistical analysis suggested that the cognitive damage was not caused by natural animal aging, which might exclude the effect of natural aging on cognitive impairment. All these results suggested that IE2 acted as a pathogenic regulator in damaging synaptic plasticity by downregulating the expression of plasticity-related proteins (PRPs), and this damage increased with aging.

## Introduction

Human cytomegalovirus (HCMV), a member of the beta-herpesviruses, has extremely high infection rates in the population all around the world (Griffiths et al., 2015). The clinical manifestations of HCMV infection include microcephaly, thrombocytopenia, jaundice, hepatomegaly, and occasionally life-threatening systemic complications, etc. (Ross and Boppana, 2005). The brain function defects caused by HCMV infection, such as intellectual disability, visual and hearing disorders, seizures, and epilepsy, have aroused great concern among researchers (Barkovich and Lindan, 1994; Grosse et al., 2008). Due to strict species-specific tropism, a HCMV-infected animal model has not been established yet. Hence, the *in vivo* mechanism of neural defects caused by HCMV remains unclear (Arvin, 2007; Compton and Feire, 2007).

Immediate early protein 2 (IE2, also known as UL122) is one of the genes first expressed after HCMV infection, which acts as a transcription factor, and is able to transcribe and activate the expression of other virus-related genes. It was found that IE2 protein disturbed brain development by the dysregulation of neural stem cell maintenance and the polarization of migrating neurons (Han et al., 2017). Our lab established a HCMV-UL122-Tg mice model (UL122 mice) which could stably express IE2 protein. Cognitive disorders were observed in the UL122 mice, and this disability was presumed to be associated with a significantly decreased expression of CX43 and NMDA receptors. Here in this research, we used the UL122 mice as a model to explore the *in vivo* influence of long-term IE2 expression on brain nerves and tried to explore other possible mechanisms.

The complexity of neurons and synaptic plasticity are thought to be the basis of memory storage (Martin et al., 2000; Martin and Morris, 2002; Rosenberg et al., 2014). The development of neurons was detected by the density of dendritic spines and branches, and the signal transmission ability between neurons was tested by long-term potentiation (LTP). Synaptic plasticity is related with several proteins. MAP2 is a kind of microtubule-associated protein, which is involved in the growth of neuronal processes and the plasticity of synapses, and is mainly distributed in the cell bodies and dendrites of neurons (Lim and Halpain, 2000; Kharlamov et al., 2009). PSD-95, as a member of the membrane-associated guanosine kinase (MAGUK) superfamily, referentially targets dendritic spines, on which it is believed to interact directly or indirectly with AMPARs and NMDAR (Kornau et al., 1997; Tomita et al., 2001). Previous studies on cultured neurons have proved that PSD-95 played an important role in synaptic maturation (Okabe et al., 2001; Prange and Murphy, 2001; El-Husseini Ael et al., 2002). The study of a PSD-95-transgenic mouse showed that PSD-95 was related to regulation of synaptic plasticity in the central nervous system (Migaud et al., 1998).

Maintaining the morphological and functional changes of synapses is important for synaptic plasticity. These changes are essential for the building and consolidation of LTP, and translation regulation is crucial to this form of memory (Chao et al., 2013; Ford et al., 2019). The RNA-binding protein commands specific PRP synthesis in neurons by regulating the transport, translation or stability of RNA, and when gene mutation or ablation might lead to abnormal memory performance in humans and mice (Costa-Mattioli et al., 2009; Darnell and Richter, 2012; Gal-Ben-Ari et al., 2012). Previous research has shown that CPEB3, as one of the cytoplasmic polyadenylation element binding protein (CPEB) family of RNA binding proteins in vertebrates, repressed the translation of several PRP RNAs, which encode for epidermal growth factor receptor and the subunits of AMPA receptor, PSD95 and NMDARs (Huang et al., 2006; Pavlopoulos et al., 2011; Chao et al., 2013). In our study, the expression of CPEB3 was increased in the hippocampal CA1 region of UL122 mice, where the expression of MAP2 and PSD95 were decreased. It was hypothesized that the increased expression of CPEB3 in the hippocampal CA1 region would inhibit the transcription and expression of MAP2 and PSD95, which could ultimately lead to reduced synaptic plasticity in the UL122 mice.

In this study, we showed that the long-term expression of IE2 protein had a profound negative impact on the cognitive ability in UL122 mice. *In vivo* expression of IE2 protein induced the abnormal development of neurons and damaged synaptic plasticity. Furthermore, the accumulation of IE2 protein in UL122 mice triggered worse behavior. Our study provides insight into the damage of long-term infection of HCMV in the neural organ.

## Materials and Methods

### Animals

The establishment of HCMV-UL122-Tg mice was described in previous publications (Jiang et al., 2015; Wang et al., 2018). HCMV-UL122-Tg mice have a C57BL/6 genetic background and were housed in an SPF level laboratory at the Experimental Animal Center of Qingdao University on a 12 h light/dark cycle with water and food available *ad libitum*. UL122 mice were detected by PCR using the following primers: S (5′- CAGTCCGCCCTGAGCAAAGA -3′) and AS (5′- TATGAACAAACGACCCAACACCC -3′). The conditions for the PCR cycle were as follows: Firstly, pre-denaturation at 94°C for 5 min; secondly, denaturation at 94°C for 30 s; annealing at 60°C for 35 s; extension at 72°C for 1 min, this circulation was performed for 35 times. At last, further extension at 72°C for 10 min was performed. We used 6-month-old UL122 mice and 12-month-old UL122 mice and separated them into adult and old groups, respectively. Depending on the presence or absence of IE2, mice were divided into the experimental group (UL122) and control group (WT). The UL122 mice and WT mice were littermates. All the animal experiments were conducted with strict adherence to guidelines and were authorized by the Animal Experiments Committee of Qingdao University.

### Morris Water Maze Assessment

Mice were trained to swim to the platform in the pool (diameter: 130 cm, depth: 60 cm; temperature: 25 ± 3°C), which was in a different location on each trial and visible for mice to recognize. After 2 days of training (5 trials per day), mice were able to reach the platform in a short time. Then the Morris Water Maze (MWM) test started on the following day. The platform was located in the center of the target zone and positioned below the surface so that mice had to search for the platform by spatial memory. At the beginning of each trail, the mouse was placed at the edge of the pool, facing the wall. The time required to move from four equidistant positions to the platform on the water surface (escape latency) was recorded. And if the mice did not arrive at the platform within 60 s, they were gently guided to the platform and allowed to stay on the platform for 10 s. Escape time was recorded as the maximum time (60 s). The learning and memory abilities of the mice were expressed as their escape latencies. Mice were examined for 6 days including 5 days for orientation navigation and the last day for space exploration trial. All the measurements were recorded using a camera with an infrared source.

### Nissl Staining

Brain tissues were removed from mice that had been administered general anesthesia and post-fixed for at least 24 h at 4°C. Then brains were sliced into 20-μm-thick coronal sections at −20°C with a cryostat (CM3050S-3, Leica Microsystems, United States). The sections were dehydrated in 95 and 70% ethanol for 1 min, respectively, and rinsed in tap water, then distilled water for 30 s. After staining for 3 min, the sections were slightly differentiated with 0.1% glacial acetic acid and washed with tap water. The slices were dried in an oven and then transparently sealed. The number of Nissl bodies was used to observe the morphological changes in the mouse hippocampus.

### Golgi Staining and Analysis

Glogi staining was conducted to assess the changes of spine density and complexity of dendrites between WT and UL122 mice. The mouse brains were dissected into 2–3-nm-thick tissue blocks and gently rinsed with normal saline several times. After being immersed in a tube added with Golgi dye for 14 days, slices were treated with 80% glacial acetic acid and analyzed by microscope image acquisition. The Eclipse CI-L photographic microscope was used to select the target area of brain tissue for 200- and 1,000-fold imaging. When imaging under the microscope, the background light of each sheet was consistent for each sheet. When imaging was completed, Image-Pro Plus 6.0 was used to measure the spine density. The number of dendritic spines in the 30–90 um length range of the second or third dendritic branches was measured at high power (1,000×), and the length of dendritic branches measured was recorded. Spine density was expressed as spines/10 μm. The Image J software was used to reconstruct all dendritic branches, and then dendritic tracking was quantified by Sholl analysis. Ten concentric circles with 10 μm spaces between them were centered on the neuron body, and the number of intersections to reflect the complexity of dendrites was counted.

### Immunohistochemistry

Tissue samples were fixed and sectioned, and the tissue sections were then treated according to a previously reported method (Xu et al., 2017). Sections were placed on a slide warmer at 70°C for 1 h, then they were deparaffinized, rehydrated, and soaked in boiling water diluted with sodium citrate buffer (pH 6.0) for 2 min to repair the antigen. After cooling to room temperature, the sections were washed with distilled water and PBS for 15 min, 3% hydrogen peroxide was used to inactivate the endogenous catalase. Sections were then incubated for 1 h with the primary antibody at 37°C (anti-MAP2, anti-PSD95, and anti-CPEB3 antibodies diluted at 1:300, 1:200, and 1:300, respectively, Bioss). After being incubated with secondary antibodies for 40 min, the sections were then exposed to DAB (Diaminobenzidine) for 1 min and then hematoxylin for 2 s, and finally observed after being sealed by neutral gum. Semi-quantitative analysis of immunohistochemistry was carried out by Image-Pro Plus 6.0. Three fields (×400) were selected randomly for each section and images were acquired. All images were acquired under the same white balance settings and exposure time. The blank area of the image was chosen for optical density correction, and the brown area was regarded as the positive expression of MAP2, PSD95, and CPEB3. The intensity of protein expression was calculated by integrated optical density (IOD) and target area (Area). Finally, the mean density (MD, IOD/Area) was used to represent the related expression of the protein.

### Immunofluorescence

The paraffin sections were dewaxed to water for antigen repair. After sealing with 3% hydrogen peroxide, the sections were sealed with serum for 30 min. First the antibody was added and left at 4°C overnight, and the corresponding labeled secondary antibody was incubated at room temperature for 50 min (anti-MAP2, anti-PSD95, and anti-CPEB3 antibodies diluted at 1:300, 1:200, and 1:300, respectively, Bioss). After the antigen was repaired, the second antibody was added to the section. Finally, nuclei were stained by DAPI and placed under the scanner to collect images after being sealed.

### Electrophysiological Recordings of Hippocampal Brain Slices

Mice were sacrificed by decapitation after a short period of inhalation of anesthetic diethyl ether. Whole brains were rapidly removed and immersed in ice-cold medium containing (in mM): Sucrose 213, KCl 3, NaH_2_PO_4_ 1, MgCl_2_ 5, CaCl_2_ 0.5, NaHCO_3_ 26, and glucose 10. The hippocampus was cut into 400-μm-thick coronal slices with a vibratome (VT-1200, Leica, Germany), and the brain slices were quickly transferred and incubated in an artificial cerebrospinal fluid (ACSF) containing (in mM): NaCl 125, KCl 5, NaH2PO_4_ 1.2, MgCl_2_ 1.3, CaCl_2_ 2.6, NaHCO_3_ 26, and glucose 10, saturated with 95% O_2_/5% CO_2_ at room temperature for 1 h prior to recording.

A hippocampal brain slice was transferred to the recording chamber and incubated with a flow of ACSF at a rate of 2 mL/min. Field excitatory postsynaptic potentials (fEPSPs) were evoked every 20 s with a bipolar platinum electrode (FHC, United States) placed in the stratum radiatum through stimulation of the Schaffer collateral pathway. Prior to every experiment, an I–O curve ranging from the threshold to the maximum response level was generated to select an appropriate stimulus intensity. The stimulus intensity was adjusted to evoke field excitatory postsynaptic potentials (fEPSPs) at half maximal amplitude, and was used throughout the measurements. The fEPSPs were monitored to be stable for 20 min as baseline recordings before a high-frequency train of stimuli (100 Hz for 1 s) was applied, and fEPSPs were recorded for another 60 min. The average slope of fEPSP at baseline was set at 100%, and changes in slope were expressed as a percentage change from the baseline.

Paired-pulse facilitation (PPF) in the CA1 region of the mouse hippocampus was obtained by giving a paired pulse with an interstimulus interval from 20 to 900 ms. The PPF was recorded in response to various interpulse intervals (20, 40, 60, 80, 100, 300, 500, 700, and 900 ms). The facilitation ratio was calculated by the second pulse-evoked fEPSP divided by the first one.

### Statistical Analysis

All the data were analyzed using SPSS software version 24.0 (SPSS Inc., Chicago, Illinois, United States). Data are presented as means ± SEM. The statistical analysis of orientation navigation statistics was performed using analysis of variance of repeated measure. Other behavioral experiments were analyzed with two-way analysis of variance (ANOVA). Immunohistochemistry and quantification of the number of dendritic branches were analyzed using *t*-test analysis. The LTP test was analyzed using one-way analysis of variance and two-way analysis of variance. *P*-values <0.05 were considered statistically significant.

## Results

### Generation of UL122 Mice

**FIGURE 1:**
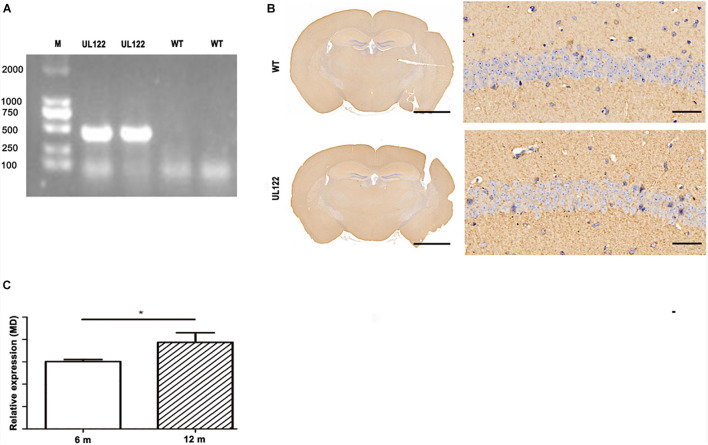
Results of PCR and immunohistochemistry in UL122 mice. **(A)** PCR result in the hippocampus of mice. Transgene PCR product size: 335 bp. **(B)** IE2 immunohistochemistry result of the hippocampal CA1 region. **(C)** The expression of IE2 in the L122 mice of different ages (6 and 12 months old). WT: The CA1 region of WT mice. UL122: The CA1 region of UL122 mice. Scale bar on the left is 2,000 μm, scale bar on the right is 50 μm.

As shown in Figure 1, the results of PCR and immunohistochemistry suggested that the UL122 mice model was successfully established (Figure 1). The result of the PCR test showed that the UL122 gene was identified from the UL122 mice (Figure 1A); the expression of IE2 was observed in the hippocampus of 6-month-old UL122 mice by immunohistochemistry (Figure 1B). Depending on the presence or absence of IE2, UL122 mice were used as the experimental group and WT as the control group, which had the same age, weight, and gender as the experimental group. The expression of IE2 was higher in 12-month-old UL122 mice than that in 6-month-old ones (Figure 1C).

### The Promotional Effect of Immediate Early Protein 2 on Cognitive Impairment Was Enhanced With Aging

**FIGURE 2:**
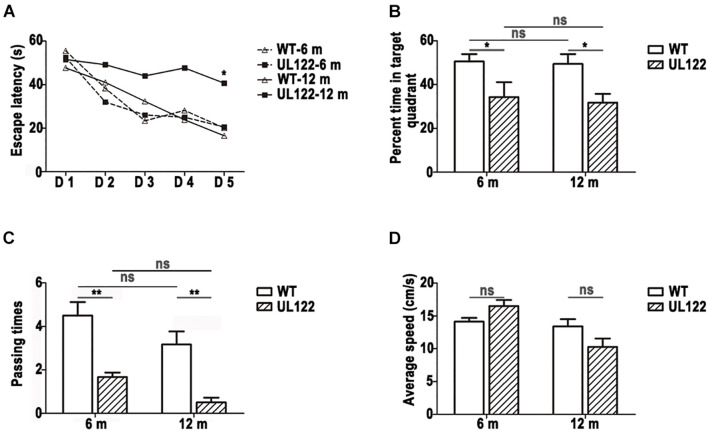
The learning and memory ability of UL122 mice aged 6 and 12 months were tested in the MWM test **(A–D)**; **(A)** The time of escaping latency. **(B)** Percentage of time in the target quadrant. **(C)** Times of passing through platform. **(D)** Average speed. Data were expressed as mean ± SEM(*n* = 6 for each group). Comparison **(A)** was made by analysis of variance of repeated measure and the comparison of the rest was analyzed with two-way analysis of variance (ANOVA) (*P <0.05, **P <0.01 vs. the control).

Due to cognitive ability decreasing with aging, the changes of cognitive function across time must also be considered. Therefore, the cognitive ability of mice aged 6 and 12 months was measure by the MWM test. According to the results, both UL122 and WT mice could learn the location of the platform evidenced by decreased latency time over the 5-day training (Figure 2A; mice aged 6 months: *P* < 0.001; mice aged 12 months: *P* < 0.01). No differences were observed between UL122 and WT mice aged 6 months in spatial learning [Figure 2A; WT mice: 32.53 ± 2.00 s; UL122 mice: 32.63 ± 2.78 s, *F*_(1, 5)_ = 0.003, *P* = 0.96], while the learning ability of UL122 mice aged 12 months was worse than WT mice aged 12 months [Figure 2A; WT mice: 32.27 ± 2.99 s; UL122 mice: 46.60 ± 2.52 s, *F*_(1, 5)_ = 9.36, *P* < 0.05]. In the probe trial, the UL122 mice aged 6 and 12 months both showed worse spatial memory for a smaller percentage of time spent in the target quadrant [Figure 2B; 6 m: 34.24% ± 6.81%; 12 months: 31.73% ± 4.03%, *F*_(1, 20)_ (12.39, P < 0.001)] and platform crossing [(Figure 2C; 6 months: 1.67 (0.21; 12 months: 0.50 (0.22, F (1, 20) (36.06, P < 0.001)] compared with the control groups. The average speed between the two groups had no significant differences [(Figure 2D; *F*_(1, 20)_ = 0.15, *P* = 0.70].

### Neuron Loss and Decreased Synaptic Plasticity in the Hippocampal CA1 Region of UL122 Mice Aged 12 Months

**FIGURE 3:**
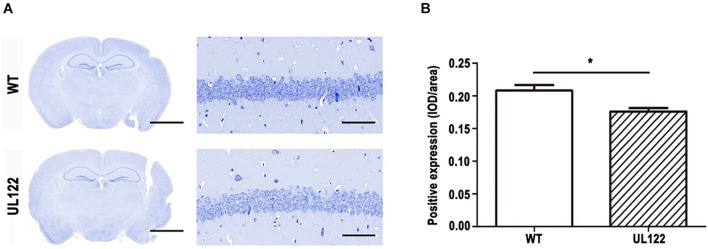
Nissl staining in the hippocampal CA1 region of UL122 mice aged 12 months. **(A)** Nissl staining of the hippocampal CA1 region of mice aged 12 months. **(B)** Quantification of Nissl staining. Data were presented as mean ± SEM(*n* = 5 for each group). Statistical analysis was performed by unpaired *t*-test. *P < 0.05 vs. the control group. Scale bar on the left is 2,000 μm, scale bar on the right is 50 μm.

**FIGURE 4:**
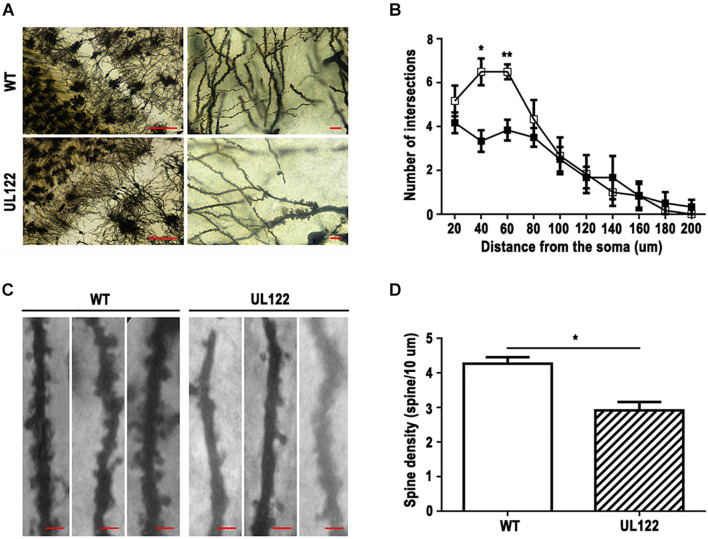
Golgi staining in the hippocampal CA1 region of UL122 mice aged 12 months. **(A,B)** Representative Golgi-Cox staining of the hippocampal CA1 region (scale bar on the left: 100 μm, scale bar on the right: 10 μm) and quantification of the number of dendritic branches. **(C,D)** Representative Golgi-Cox staining (scale bar: 10 μm) and quantification of the dendritic spine density. All data were presented as mean ± SEM(*n* = 10 neurons per mouse, 6 mice per group). Statistical analysis was performed by unpaired *t*-test. *P < 0.05, **P < 0.01 vs. the control group.

To explore the causes of learning and memory impairment in the UL122 mice aged 12 months at the organizational level, number of neurons, number of neuronal dendritic branches, and spine density were measured by Nissl staining and Golgi staining. Nissl staining results showed that the number of neurons in the hippocampal CA1 region of UL122 mice was significantly lower than that in the control group (Figure 3). As shown in Figure 4, the mean number of neuronal dendritic branches and spine density were significantly decreased in the hippocampus of UL122 mice compared with control mice (^∗^*P* <0.05, ^∗∗^*P* <0.01). These results suggested that expression of IE2 might led to the decrease in the number of neurons and impairment of synaptic plasticity of UL122 mice aged 12 months.

### Long-Term Potentiation Impairment and Basal Synaptic Transmission at the SC-CA1 Pathway in UL122 Mice Aged 12 Months

**FIGURE 5:**
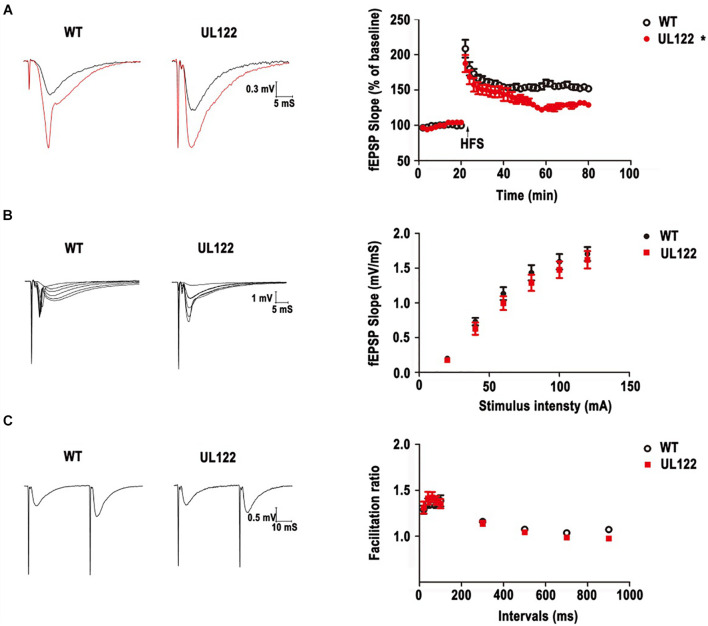
LTP impairment and basal synaptic transmission in WT mice and UL122 mice. **(A)** Representative traces before (black line) and after (red line) LTP inductions. HFS–induced LTP in hippocampal CA1 neurons from WT mice. *P < 0.05 vs. WT group. **(B)** The input-output curves at the SC-CA1 pathway in hippocampal slices from WT mice and UL122 mice. *n* = 10/8 (10 slices from 8 mice) for WT mice, *n* = 11/8 (11 slices from 8 mice) for UL122 mice. **(C)** Paired-pulse facilitation at the SC-CA1 pathway in hippocampal slices from WT mice and UL122 mice. *n* = 10/8 for WT mice, *n* = 11/8 for UL122 mice. Data were expressed as mean ± SEM. Comparison was made by two-way or one-way analysis of variance. *The slope of EPSPs in the maintaining stage of UL122 mice group was significantly decreased than that of WT mice.

Based on the morphological damage and quantity reduction of neurons (Figures 3, 4), the LTP in the hippocampal CA1 region was recorded to assess the impairment of hippocampal synaptic functional plasticity in UL122 mice aged 12 months compared with the control after the behavior test. As shown in Figure 5A, after 20 min of baseline recording, LTP was significant induced by giving a high frequency simulation (HFS) in both groups. Nevertheless, LTP was significantly impaired in the maintaining stage in the group of UL122 mice (129.0 ± 1.6) compared with control (151.9 ± 2.3%). These data indicated that LTP might be impaired in UL122 mice aged 12 months.

We further investigated the postsynaptic activity with increasing stimulus intensity. As shown in Figure 5B, the slope of EPSPs was also increased with increasing tetanization (0.02–0.2 mA) in each group. However, the input-output curves showed a decrease tendency in the UL122 group, but without a significant difference between the two groups.

Then PPF was examined to test presynaptic plasticity by giving a short interpulse interval (20∼900 ms) on brain slices from the hippocampal CA1 area. As shown in Figure 5C, the facilitation ratio was determined by evoking two fEPSPs and dividing the initial slope of the second fEPSP by that of the first (fEPSP2/fEPSP1). There was no differences or reduced tendency of UL122 mice compared with control mice, indicating that there was no alteration of presynaptic properties. Taken together, these results suggest that basal synaptic transmission does not change in UL122 mice aged 12 months.

### Expression of Microtubule-Associated Protein 2 and Postsynaptic Density Protein 95 in the Hippocampal CA1 Region of UL122 Mice Aged 6 Months and 12 Months

**FIGURE 6:**
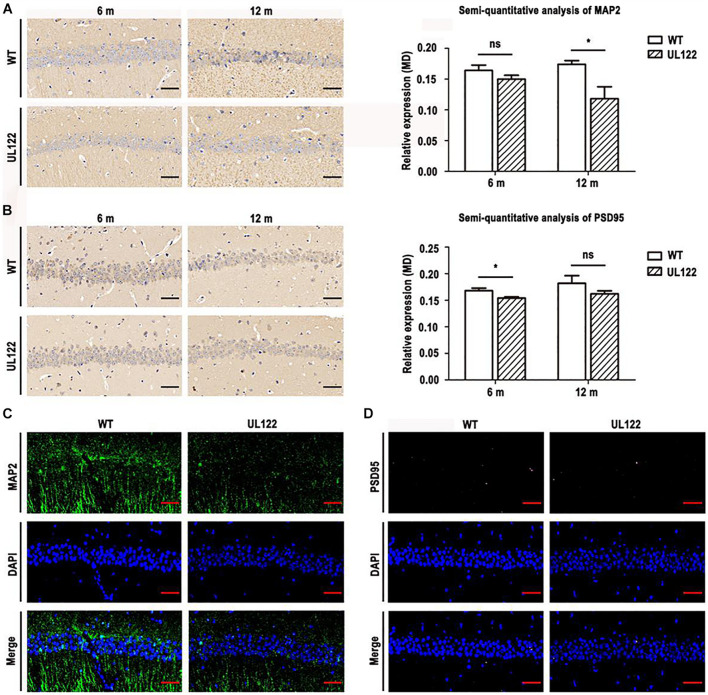
The expression of MAP2 and PSD95 in the hippocampal CA1 region of mice aged 6 and 12 months. **(A)** MAP2 immunohistochemistry and semi-quantitative analysis of immunohistochemistry for MAP2. **(B)** PSD95 immunohistochemistry and semi-quantitative analysis of immunohistochemistry for PSD95. **(C)** The expression of MAP2 and PSD95 in the CA1 region of mice aged 12 months measured by immunofluorescence. **(D)** The expression of PSD95 in the CA1 region of mice aged 12 months measured by immunofluorescence. MAP2 detected with a primary monoclonal antibody (green), PSD95 detected with a primary monoclonal antibody (pink), nuclei stained with DAPI (blue), merged mage. The bars indicate the means ± SEM(*n* = 4 for each group). Statistical analysis was performed by unpaired *t*-test. *P <0.05: significantly different from the WT group.

It is well known that MAP2 and PSD95 play an important role in maintaining synaptic morphological and functional plasticity. In our study, MWM and LTP test results showed that synaptic plasticity in the UL122 mice was impaired. Therefore, the expression of PRPs was detected to analyze the molecular mechanism of synaptic plasticity damage. As shown in Figure 6, the PRPs including MAP2 and PSD95 in the hippocampal CA1 region at two different periods after birth were measured. As shown in Figure 6A, the expression of MAP2 in the UL122 group was significantly lower than that in the control group aged 12 months (^∗^*P* <0.05). It suggested that the decreased expression of MAP2 in the hippocampal neurons of UL122 mice might be caused by IE2. Besides, the expression of PSD95 in UL122 mice was significantly lower than that in the control group aged 6 months (Figure 6A). However, there were no great differences between the two groups at 12 months (Figure 6B). Immunofluorescence showed that the expression of MAP2 in the UL122 mice was significantly lower than that in control mice aged 6 months (Figure 6C), while there was no difference in the expression of PSD95 between the two groups aged 12 months (Figure 6D). These results suggested that the damage of synaptic plasticity was related to IE2 downregulating PSD95 in the UL122 mice aged 6 months. Besides, the damage of synaptic plasticity in the UL122 mice aged 12 months might be related to the expression of MAP2 rather than PSD95.

### CPEB3 Was Upregulated in the Hippocampal CA1 Region of UL122 Mice

**FIGURE 7:**
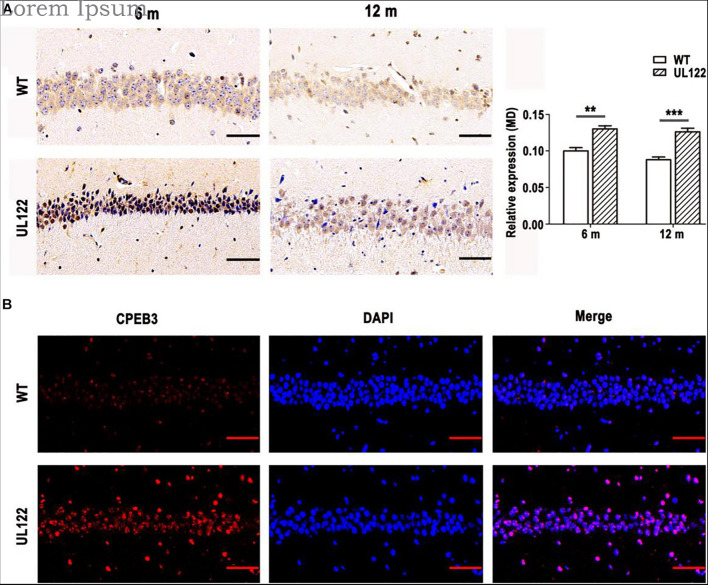
The expression of CPEB3 in the hippocampal CA1 region of mice aged 6 and 12 months. **(A)** CPEB3 expressed in the hippocampal CA1 region. CPEB3 immunohistochemistry **(B)**. The expression of CPEB3 in the hippocampal CA1 region was measured by immunofluorescence. The bars indicate the means ± SEM (*n* = 4 for each group). Statistical analysis was performed by unpaired *t*-test. ***P < 0.001, **P < 0.005: significantly different from the WT group. Bar: 50 μm.

Previous studies have shown that PRPs could be downregulated by CPEB3 *in vivo* (Matus, 1988; Halpain and Greengard, 1990; Johnson et al., 1991; Martin and Morris, 2002). To further determine whether CPEB3 affected the expression of MAP2 and PSD95 in the hippocampal neurons of UL122 mice, the expression of CPEB3 in the hippocampal CA1 region was measured. As shown in Figure 7A, the expression of CPEB3 between UL122 mice aged 6 months and 12 months was significantly different compared with the control mice. As the expression of CPEB3 in UL122 mice was negatively correlated with the expression of MAP2 and PSD95 (Figure 6), it might be speculated that the mechanisms underlying decreased expression of PRPs in the UL122 mice might be related to the elevated expression of CPEB3. Besides, cognitive impairment in the UL122 mice aged 6 months was related to the decreased expression of PSD95, however the cognitive impairment in the UL122 mice aged 12 months was associated with decreased expression of MAP2 rather than PSD95, which might explain the mechanism of HCMV infection promoting cognitive impairment in the elderly.

## Discussion

UL122 (also named as IE2/IE86), the major immediate-early protein of HCMV, is critical for efficient viral replication. UL122 has been reported to play a key role in HCMV-induced cognitive impairment. Our research team had established HCMV-UL122-Tg mice (UL122 mice) as an animal model to explore the function of UL122. In our previous research, UL122 mice displayed significant cognitive impairment compared with the control group, and this appearance was related to the abnormal expression of several genes including Cx43, NMDA, fibroblast growth factor 2 (FGF2), neuron-specific nuclear protein (NeuN), glial fibrillary acidic protein (GFAP), SYP, and PSD-95. This study was conducted to detect whether cognitive impairment aggravates with age. The older UL122 mice (12 months old) were more likely to develop cognitive dysfunction compared with adult UL122 mice (6 months old), reflected by poor performance in the MWM and LTP tests. Reduced density of dendritic spines and branches was detected in UL122 mice by Golgi staining. Moreover, aged UL122 mice showed decreased PRPs (PSD95 and MAP2) levels, which may cause the impairment of synaptic plasticity. The results of transmission electron microscopy were also consistent with these results (Supplementary Figure 1). And the level of CPEB3, which could downregulate PRPs expression, was increased in UL122 mice. All these results might suggest a hypothesis that IE2 causes cognitive disorder in UL122 mice by upregulating CPEB3 and therefore downregulating PRPs, that results in the impairment of synaptic plasticity and development of dendritic cells.

Compared with 6-month-old mice, the 12-month-old UL122 mice performed worse in the MWM test. And the synaptic plasticity and development of dendritic cells results were consistent with this conclusion. The influence of age itself was excluded by statistical analysis. And results in Figure 1C show that the expression of IE2 was higher in 12-month-old mice than in 6-month-old ones. On the one hand, these results suggested that the continuing accumulation of IE2 damaged neuro cells and resulted in serious cognitive behavior impairment. On the other hand, in order to establish a better intracellular environment for replication, IE2 might interfere with the immune-associated pathways. Hence, the changes of the immune microenvironment are highly likely to contribute to the expression of IE2.

Studies have confirmed that MAP2 combined with microtubules and provided support for the cytoskeleton of neurons, which was important for the stability of the dendritic structure and axons growth (Sanchez et al., 2000; Dehmelt and Halpain, 2005). In addition, MAP2 is essential for cell functions such as local signal transduction, protein transport, integration of synaptic inputs, and synaptic plasticity (Urbanska et al., 2008; Gardiner et al., 2011). PSD95, as a member of the membrane-associated guanosine kinase (MAGUK) family, is one of the most abundant proteins of PSD (postsynaptic density), and also plays an important role in glutamate transmission, dendritic spine morphogenesis, and synaptic plasticity in the process of neural development (Kim and Sheng, 2004; Funke et al., 2005; Gilman et al., 2011). As a sequence-specific RNA-binding protein, CPEB3 limits the strength of glutamatergic synapses by downregulating the expression of multiple plasticity-related proteins (PRPs), such as N-methyl-D-aspartate receptor (NMDAR) and PSD95, etc. Our previous studies have shown that IE2 could reduce the expression of NR1 in the hippocampal CA1 region of UL122 mice. Immunohistochemistry results showed that the expression of CPEB3 in the hippocampal CA1 region was significantly increased in the UL122 mice (Figure 7). The LTP test has shown that the synaptic plasticity of the hippocampal CA1 region was significantly impaired (Figure 5). As PRPs reduced CPEB3 elevated simultaneously in UL122 mice, and in our previous publications, the expression of NR1 was abnormal in UL122 mouse (Wang et al., 2018), it could be proposed that IE2-CPEB3-mediated PRPs might represent a novel mechanism underlying the cognitive impairment in the UL122 mice.

The cognitive disorder in UL122 mice might be related to its function as the regulator of a number of genes. Previous research has confirmed that IE2 could attenuate the expression of beta interferon (IFN-β) and a number of pro-inflammatory chemokines, via inhibiting virus-induced binding of NF-κB to the IFN-β promoter (Taylor and Bresnahan, 2006). This suppression of cytokine and chemokine gene expression during HCMV infection may provide a favorable cellular environment for viral replication and persistence. Previous results suggested that truncated forms of the IE2 protein interacted with TBP, TFIIB, and TAFII130/TAF4 *in vitro*, and the IE2 protein rescued defective TAFII250 (Lee et al., 2020). Other cellular transcription factors and chromatin remodeling proteins could also interact and contribute to the activity of the IE2 protein, such as CREB, SP1, Tef-1, Egr-1, p300/CBP, and P/CAF (Lukac et al., 1994; Sommer et al., 1994; Lang et al., 1995; Bryant et al., 2000). IE2 mediates the stimulator of interferon gene degradation and blocks the cGAS-STING pathway (Lee et al., 2020). The continuing expression of IE2 might be involved in the disorder binding of several genes. Since the direct interactions of IE2 and neuro development-related genes have not been reported, this research might help by offering new insights on exploring genes affected by IE2.

## Conclusion

In conclusion, this research suggests that there might be a mechanism to modulate the expression of PRPs (PSD95 and MAP2). In the UL122 mice, the increased expression of CPEB3 induced by long-term stably expressed IE2 impaired synaptic plasticity through downregulating the expression of PRPs (PSD95 and MAP2), finally resulting in cognitive impairment. It is possible that other PRPs might also contribute to the impaired synaptic plasticity, which will be investigated in our follow-up research work.

## Data Availability Statement

The original contributions presented in the study are included in the article/Supplementary Material, further inquiries can be directed to the corresponding author.

## Ethics Statement

The animal study was reviewed and approved by Animal Experiments Committee of Qingdao University.

## Author Contributions

BW, ZW, and ZT conceived and designed the experiments, contributed reagents, materials, and analysis tools, wrote the manuscript, and processed the figures. YW and LL modified the manuscript. WY, JN, XZ, DK, LX, BJ, and WZ performed the experiments and analyzed the data. All authors were involved in critical revision, final approval of the manuscript for publication, and agreed to be accountable for all aspects of the work in ensuring that questions related to the accuracy or integrity of any part of the work are appropriately investigated and resolved.

## Conflict of Interest

The authors declare that the research was conducted in the absence of any commercial or financial relationships that could be construed as a potential conflict of interest.

## Publisher’s Note

All claims expressed in this article are solely those of the authors and do not necessarily represent those of their affiliated organizations, or those of the publisher, the editors and the reviewers. Any product that may be evaluated in this article, or claim that may be made by its manufacturer, is not guaranteed or endorsed by the publisher.
