# Supplementary material for: Human Cytomegalovirus Immediate Early Protein 2 Protein Causes Cognitive Disorder by Damaging Synaptic Plasticity in Human Cytomegalovirus-UL122-Tg Mice
Source: Front Aging Neurosci. 2021 Nov 1;13:720582. doi: 10.3389/fnagi.2021.720582 (PMC8591137; doi:10.3389/fnagi.2021.720582)
Supplement: Supplementary file 1 [file Data_Sheet_1.doc]

Supplementary Figure 1: Decreased postsynaptic density in the aged UL122 mice. As shown in Supplementary Figure 1, the thickness of postsynaptic densities in the group of UL122 mice (31.1 ± 3.6 nm) were significantly thinner than in the control (46.0 ± 1.68 nm).


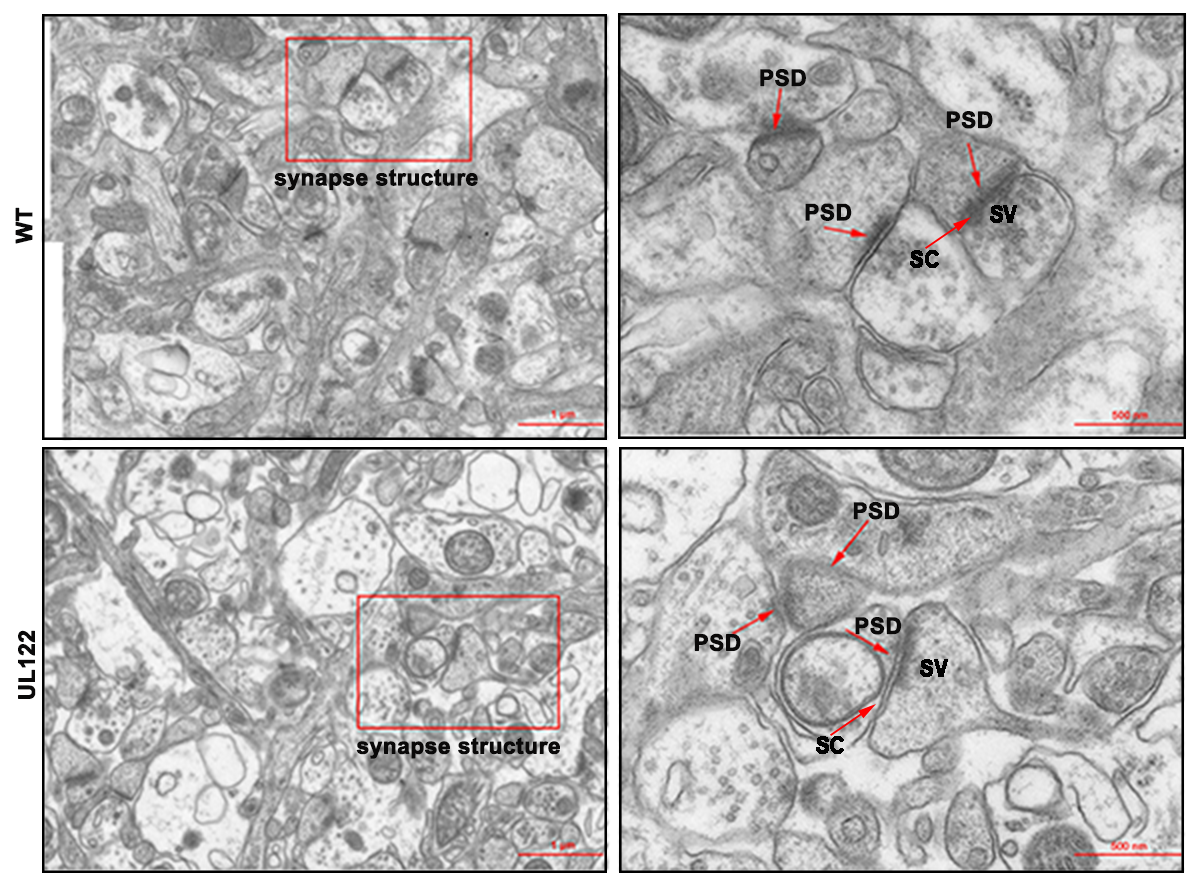


Supplementary Figure 1. Effects of UL122 on postsynaptic density of hippocampal CA1 region in UL122 mice. PSD, postsynaptic density; SV, synaptic vesicle; SC, synaptic cleft; WT represents a 12-month-old WT mouse, UL122 represents a 12-month-old UL122 mouse. Slice thickness=80 nm. Data were expressed as mean ± SEM (nm), n=3, left scale bar=1 µm; right scale bar=500 nm.

Supplementary Figure 2: Expression of IE2 protein in the hippocampus and cortex


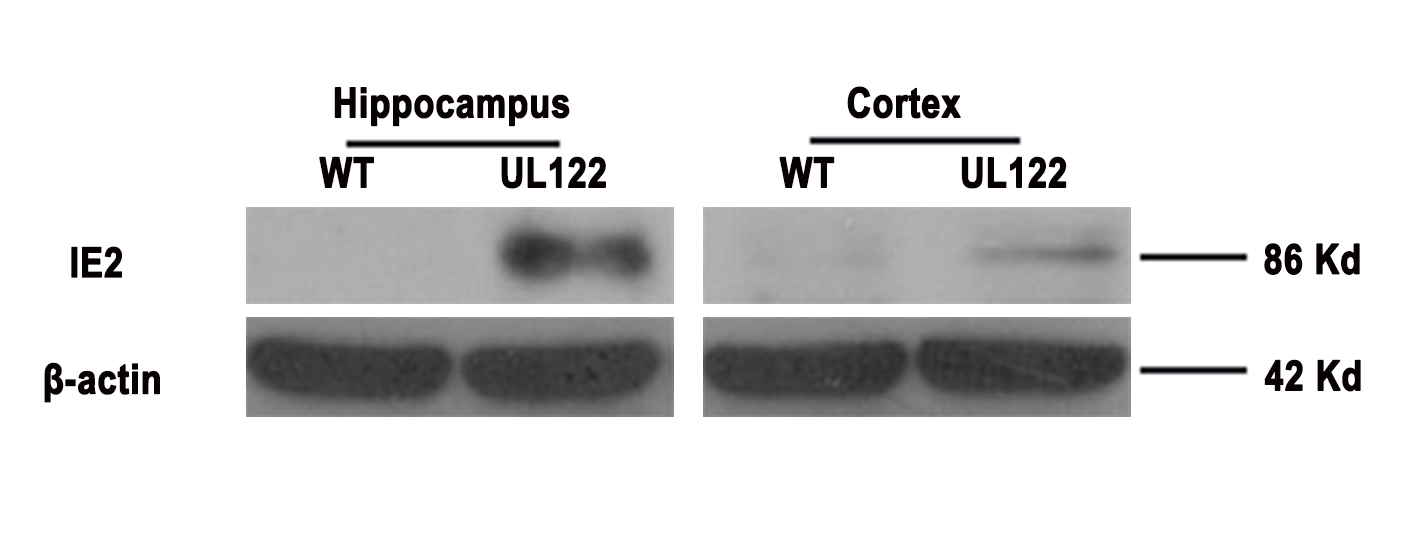


Supplementary Figure 2. The expression levels of IE2 in the hippocampus and cortex are different. WT represents a 6-month-old WT mouse, UL122 represents a 6-month-old UL122 mouse. IE2: 86 Kd, β-actin: 42 Kd.
